# Galanin regulates blood glucose level in the zebrafish: a morphological and functional study

**DOI:** 10.1007/s00418-015-1376-5

**Published:** 2015-10-26

**Authors:** P. Podlasz, A. Jakimiuk, M. Chmielewska-Krzesinska, N. Kasica, N. Nowik, J. Kaleczyc

**Affiliations:** Department of Animal Anatomy, Faculty of Veterinary Medicine, University of Warmia and Mazury, Oczapowskiego 13, 10-719 Olsztyn, Poland; Department of Pathophysiology, Forensic Veterinary and Administration, Faculty of Veterinary Medicine, University of Warmia and Mazury, Oczapowskiego 13, 10-719 Olsztyn, Poland

**Keywords:** Galanin, Blood glucose, Insulin, Somatostatin, Parasympathetic ganglia, Zebrafish

## Abstract

The present study has demonstrated the galaninergic innervation of the endocrine pancreas including sources of the galaninergic nerve fibers, and the influence of galanin receptor agonists on blood glucose level in the zebrafish. For the first time, a very abundant galaninergic innervation of the endocrine pancreas during development is shown, from the second day post-fertilization to adulthood. The fibers originated from ganglia consisting of galanin-IR, non-adrenergic (non-sensory) neurons located rostrally to the pancreatic tissue. The ganglia were found on the dorsal side of the initial part of the anterior intestinal segment, close to the intestinal branch of the vagus nerve. The galanin-IR neurons did not show immunoreactivity for applied antibodies against tyrosine hydroxylase, choline acetyltransferase, and vesicular acetylcholine transporter. Intraperitoneal injections of galanin analog NAX 5055 resulted in a statistically significant increase in the blood glucose level. Injections of another galanin receptor agonist, galnon, also caused a rise in blood glucose level; however, it was not statistically significant. The present findings suggest that, like in mammals, in the zebrafish galanin is involved in the regulation of blood glucose level. However, further studies are needed to elucidate the exact mechanism of the galanin action.

## Introduction

Galanin is a 29- to 30-amino acid neuropeptide widely expressed in the central and peripheral nervous systems (Ch’ng et al. 1985; Melander et al. 1986; Podlasz et al. 2012; Skofitsch and Jacobowitz 1985). It is coexpressed in neurons with several small molecular classical neurotransmitters (Mazarati et al. 2000) and exerts strong inhibitory action on synaptic transmission by reducing their release (Hokfelt et al. 1998; Misane et al. 1998; Pieribone et al. 1995; Zini et al. 1993). The first reported biological activity of galanin was its effect on plasma glucose levels in dogs and rats (Tatemoto et al. 1983). Immunohistochemical studies have demonstrated galanin-positive nerve fibers in the pancreas in several species including humans (Ahren et al. 1991; McDonald et al. 1992; Shimosegawa et al. 1992), rat and mouse (Adeghate and Ponery 2001; Lindskog et al. 1991), dog (Dunning et al. 1986; Taborsky et al. 1999), cat (Furuzawa et al. 1996), pig (Adeghate et al. 1996; McDonald et al. 1992; Messell et al. 1990), ruminants (Baltazar et al. 2000, 2001), chicken (Hiramatsu and Ohshima 1995), lizards (Della Rossa and Putti 1995), bullfrog (Kawakami et al. 1995), and also fish (Bosi et al. 2004, 2007; Putti et al. 2000). The suppression of insulin release by galanin has been demonstrated in dogs (Dunning et al. 1986), and this effect is mediated by G_i_-protein (Nilsson et al. 1989). However, the function of galanin in the endocrine pancreas is not fully elucidated. There are many species-related differences regarding galaninergic innervation of the endocrine pancreas and function of galanin in the regulation of blood glucose level. For instance, in humans intravenous administration of galanin does not affect glucose-stimulated insulin secretion (Ahrén 1990; Gilbey et al. 1989), although galanin inhibits glucose-stimulated insulin secretion from the isolated human islets (Ahren et al. 1991). Moreover, galaninergic fibers innervating pancreatic islets are numerous in the dog and mouse, but very rare in the rat (Ahren et al. 1990; Lindskog et al. 1991). Some studies have revealed that galanin is a sympathetic neurotransmitter in the islets (Ahren et al. 1990; Taborsky et al. 1999). However, other origins of this peptide cannot be excluded as far as glucose homeostasis is concerned.

Pancreatic islets are richly supplied with autonomic nerves. The fibers usually follow the blood vessels, penetrate into the islets, and terminate close to the endocrine cells. In mammals, the adrenergic/sympathetic axons innervating the islets are postganglionic with most nerve cell bodies located in the celiac ganglion or in the paravertebral sympathetic ganglia (Ahren et al. 1986; Brunicardi et al. 1995). The preganglionic parasympathetic fibers derive from the dorsal motor nucleus and travel within the vagus nerve. The preganglionic fibers synapse at intrapancreatic ganglia where postganglionic fibers reaching the pancreatic islets originate from (Miller 1981).

The zebrafish (*Danio rerio*) has become known as an excellent model organism for studies of vertebrate biology, particularly for those dealing with vertebrate genetics and embryonal development. It is also a potential model for human diseases. There are many zebrafish models of human diseases including genetic disorders and acquired diseases, including diabetes (Jopling et al. 2010; Kawahara et al. 2011; Olsen et al. 2010; Panula et al. 2006; Santoriello et al. 2010; Swanhart et al. 2011; Xi et al. 2011). Zebrafish have a number of features making them an attractive research tool. A fundamental advantage is that they share a considerable amount of genetic identity with humans, and several of their organ systems are remarkably similar to those found in mammals. Also, the zebrafish pancreas shares morphological and physiological similarities with the human pancreas. Many laboratories use the zebrafish to study pathological processes affecting this organ in humans (for review, see Kinkel and Prince 2009). The extensive innervation of the mammalian pancreatic islets during the ontogeny suggests that the nerves may regulate the islet development and maturation and can be a source of extrinsic factors supporting these processes (Proshchina et al. 2014). Galanin, in addition to the classic role of neurotransmitter, also has other functions. There is some evidence that it plays a role of the trophic factor during the ontogeny (Hobson et al. 2010). However, the literature in the field contains no information on the autonomic innervation of the zebrafish pancreas or on the potential influence of (one of the autonomic neurotransmitters) galanin on function of its endocrine portion. Therefore, the aim of the present study was to investigate the galaninergic innervation of the endocrine pancreas including sources of the galaninergic nerve fibers, and the influence of galanin receptor agonists on blood glucose level in the zebrafish.

## Materials and methods

### Animals

The animals were housed and treated in compliance with the rules of the local Ethics Commission (affiliated to the National Ethics Commission for Animal Experimentation, Polish Ministry of Science and Higher Education). In the study, the wild-type Tubingen and Tg(mnx1:TagRFP) zebrafish were used. The transgenic zebrafish line Tg(mnx1:TagRFP) (Jao et al. 2012) was used to localize β cells. *Mnx1* (motor neuron and pancreas homeobox 1) gene was reported to be expressed in motor neurons and pancreas. In the zebrafish pancreas, *mnx1* is expressed exclusively in β cells by 20 hpf (Wendik et al. 2004).

The adult zebrafish were reared on a 14:10-h light–dark photoperiod cycle, at 28.5 °C. Fish feeding, breeding, and maintenance were done according to Westerfield (2000). The embryos were obtained by natural mating. They were kept at 28.5 °C.

### Zebrafish galanin probe synthesis

The pGEM-T-Easy vector containing *galanin*-cDNA was linearized with either *Spe*I (antisense) or *Nco*I (sense). Digoxigenin (DIG)-labeled sense and antisense RNA probes were made using SP6 and T7 polymerase, respectively, using DIG RNA labeling kit. DIG-labeled probes were purified, quantified, and checked for integrity as described in detail elsewhere (Chen et al. 2009).

### Whole-mount in situ hybridization

The embryos were fixed in 4 % paraformaldehyde in phosphate buffer (PB), pH 7.4 (PFA), dehydrated with a series of phosphate-buffered saline + 0.1 % Tween 20 (PBSTw)–methanol solutions (3:1, 1:1, 1:3) at room temperature (RT) for 10 min each, and finally stored in 100 % methanol overnight at −20 °C. They were rehydrated with a series of PBSTw–methanol solutions (1:3, 1:1, 3:1) and digested with proteinase K. Prehybridization and the subsequent hybridization were conducted with a probe at 70 °C overnight. After hybridization, the embryos were washed in a series of the following solutions: 50 % formamide, 5× SSC, 0.1 % Tween 20 (Hyb^−^)—2× 0.15 M NaCl, 0.015 M Na citrate, 0.1 % Tween 20 (SSCT) solution (3:1, 1:1, 1:3), 2× SSCT and 0.2× SSCT—all at 70 °C for 15 min each. Next, they were washed in a series of other solutions: 0.2× SSCT–PBSTw (3:1, 1:1, 1:3), PBSTw and blocked in a blocking buffer for 3 h, followed by incubation with alkaline phosphatase-conjugated anti-DIG antibody (1:2000 dilution) in blocking solution overnight at 4 °C. The specimens were washed with PBSTw three times and then once in alkaline phosphatase buffer (100 mM Tris, pH 9.5, 50 mM MgCl_2_, 100 mM NaCl, and 0.1 % Tween 20). The embryos were allowed to develop color in HNPP/Fast Red TR mix (Roche Diagnostics) in alkaline phosphatase buffer. After they developed color, the coloring reaction was stopped by washing twice with PBSTw and then fixing in 4 % PFA in PB, pH 7.4. The embryos were analyzed with LSM 700 confocal laser scanning microscope (Zeiss, Germany). Fluorophores were excited at 555 nm by a solid-state laser.

### Immunohistochemistry and microscopy

The specimens were fixed with 4 % PFA o/n at +4 °C. The embryos and larvae were whole-mount-fixed. In the older fish, the abdomen cavity was opened before fixation and guts were dissected. The guts from the adults were cryoprotected in sucrose (20 % in 0.1 M PB) and kept for additional 24–48 h at 4 °C followed by freezing and embedding (Tissue-Tek^®^ O.C.T™ Compound, Sakura). Then, they were cryosectioned (20 µm) and the sections were collected on gelatin-coated slides. Next, the specimens were washed and preincubated in PBS containing Triton X-100 0.3 % (PBS-T, pH 7.4), 1 % dimethyl sulfoxide (DMSO), 0.1 % sodium azide, and 4 % normal goat serum (NGS) for 4–6 h at RT. The specimens were then incubated with the primary antibodies (Table 1) in the preincubation solution for 14–24 h at RT. Afterward, they were washed thoroughly with PBS-T and incubated with the secondary antibodies (Table 1) diluted 1:1000 in the preincubation solution for 12–24 h at 4 °C for whole-mount immunohistochemistry and for 1 h at RT for sections. After extensive washing with PBS and with 50 % glycerol in PBS, the specimens were mounted in 80 % glycerol in PBS. They were examined with a LSM 700 confocal laser scanning microscope (Zeiss, Germany). Stacks of images were compiled to produce maximum intensity projection images with ZEN 2009 software (Zeiss, Germany). Additionally, each optical section was analyzed separately, section by section, to detect every minor detail.

**Table 1:** Primary and secondary antibodies used in the study

Antigen	Immunogen	Host	Clonality	Dilution	Company	Catalog no.
Primary antibodies						
Galanin	Rat galanin synthetic peptide	Rabbit	Polyclonal	1:5000	Millipore	AB2233
HuC/D	Human HuC/D neuronal protein	Mouse	Monoclonal	1:1000	Invitrogen	A-21271
AcTub	Acetylated alpha-tubulin from the axoneme of sea urchin sperm flagella	Mouse	Monoclonal	1:1000	Invitrogen	32-2700
TH1	Tyrosine hydroxylase purified from PC12 cells	Mouse	Monoclonal	1:2000	Millipore	MAB318
ChAT	Human placental enzyme	Goat	Polyclonal	1:100	Millipore	AB144P
Sst	Somatostatin conjugated to thyroglobulin	Rat	Monoclonal	1:100	Serotec	8330-0009

Antigen	Fluorophore	Host	Dilution	Company	Catalog no.
Secondary antibodies					
Rabbit IgG	Alexa 635	Goat	1:1000	Invitrogen	A-31576
Mouse IgG	Alexa 488	Goat	1:1000	Invitrogen	A-11029
Rabbit IgG	Alexa 555	Goat	1:1000	Invitrogen	A-21431
Rabbit IgG	Alexa 488	Donkey	1:1000	Invitrogen	A-21206
Goat IgG	Alexa 555	Donkey	1:1000	Invitrogen	A-21432
Rat IgG	Alexa 488	Goat	1:1000	Invitrogen	A-11006

Careful control investigations for antiserum specificity were carried out. Preabsorption controls of the galanin antiserum were done by preincubating the antibody with the rat galanin peptide (Bachem, Bubendorf, Switzerland; 10 μg/ml) for 24 h under slow stirring at 4 °C. Negative controls were performed by omitting all primary or secondary antibodies in the staining protocol. All types of controls led to complete elimination of the immunostaining.

#### Intraperitoneal (IP) injections

For the functional experiment, the adult fish (12 months old, males and females) were used. Before the injections, the fish were fasted for 72 h. Weight (g) was measured by putting the animal into a small beaker of facility water on a scale and subtracting the non-fish weight.

To anesthetize the fish for IP injection, it was placed in a beaker containing 0.02 % MS-222 (tricaine; Sigma) in facility water at 28.5 °C. When the animal reached stage II of anesthesia (Brown 1993; Iwama and Ackerman 1994), it was placed on a surgical table constructed as described elsewhere (Eames et al. 2010). The sponge was cut in half, and a shallow trough was cut into the flat face. The trough was used for holding the fish securely during the injection. The surgical table with the fish was immediately transferred to an adjacent dissecting microscope stage (SteREO Discovery.V8 Stereomicroscope, Zeiss, Germany) for injection. The injection was performed using a 10-μl Hamilton syringe (Hamilton Company). The needle was inserted at the midline into the ventral posterior abdomen, between the pelvic fins. The injection site was located closer to the insertion of the fins on the pelvic girdle rather than to the anus. The needle was directed cranially to reduce the possibility of causing damage to internal organs.

The fish in the first group (*n* = 10) were injected with 15 µg/g of NAX 5055, a galanin peptide analog and non-selective galanin receptor agonist (kindly granted by Drs. Steve White and Grzegorz Bulaj from the University of Utah (White et al. 2009)), and 0.5 mg/g d-glucose (Sigma) dissolved in Cortland salt solution, pH 7.45. The animals of the second group (*n* = 10) were injected with 5 µg/g of galnon, a non-peptide, non-selective galanin receptor agonist (Bachem) and 0.5 mg/g d-glucose. The control fish (*n* = 10) were injected only with 0.5 mg/g d-glucose.

#### Whole blood glucose measurement

**Fig. 1:**
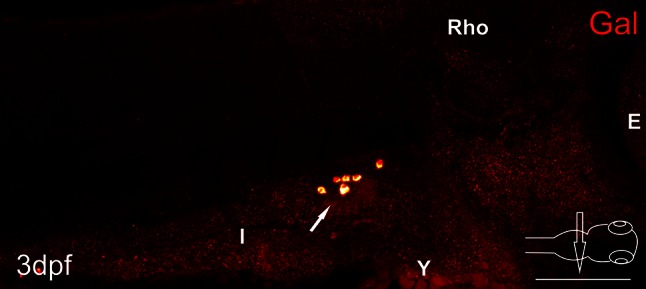
Whole-mount fluorescence in situ hybridization of galanin expression of 3 day post-fertilization (dpf) zebrafish larvae. *Arrows* point to galaninergic neurons in the autonomic ganglion. *E* eye, *I* intestine, *Rho* rhombencephalon, *Y* yolk. *Scale bar* 100 µm

The fish were anesthetized with 0.02 % MS-222, 2.5 h after the injection (tricaine; Sigma). When they had reached stage III, plane 2 of anesthesia (surgical plane) (Brown 1993; Iwama and Ackerman 1994), whole blood was collected from the caudal artery by cutting out the fish tail with scissors, and then the animals were killed by decapitation. Whole blood was analyzed immediately using OneTouch Select glucometer (LifeScan) (Fig. 1).

### Statistical analysis

The analysis was performed using GraphPad Prism, version 5.02, and one-way ANOVA test. The averages are reported as mean ± SE of the mean.

## Results

**Fig. 2:**
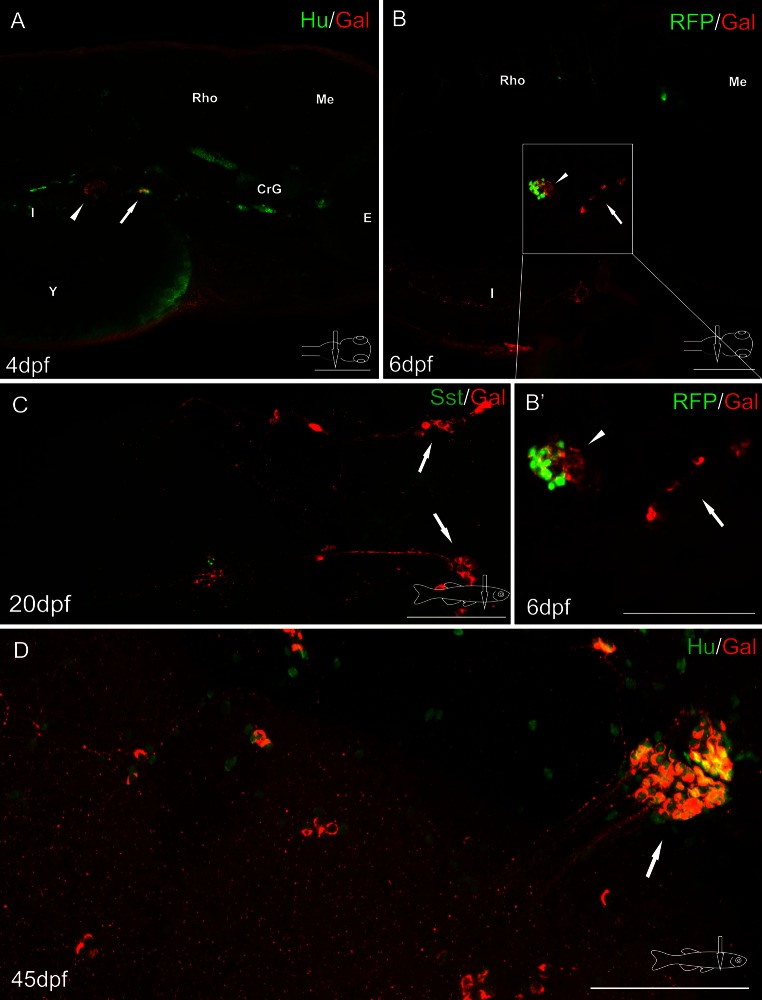
Whole-mount immunofluorescence staining of the zebrafish larvae (**a**, **b**) and juvenile zebrafish gut (**c**, **d**) using antibody against galanin (**a**–**d**) and neuronal marker Hu (**a**, **d**), somatostatin (Sst) (**c**). Red fluorescence protein (RFP) marked mnx1+ population of β cells (**b**, **b**′). *Arrows* show galaninergic neurons in the autonomic ganglion. In the primary islet of the endocrine pancreas, galanin-IR fibers formed a very dense network (*arrowheads*). *CrG* cranial ganglia, *E* eye, *I* intestine, *Me* mesencephalon, *Rho* rhombencephalon, *Y* yolk. *Scale bars* 100 µm

**Fig. 3:**
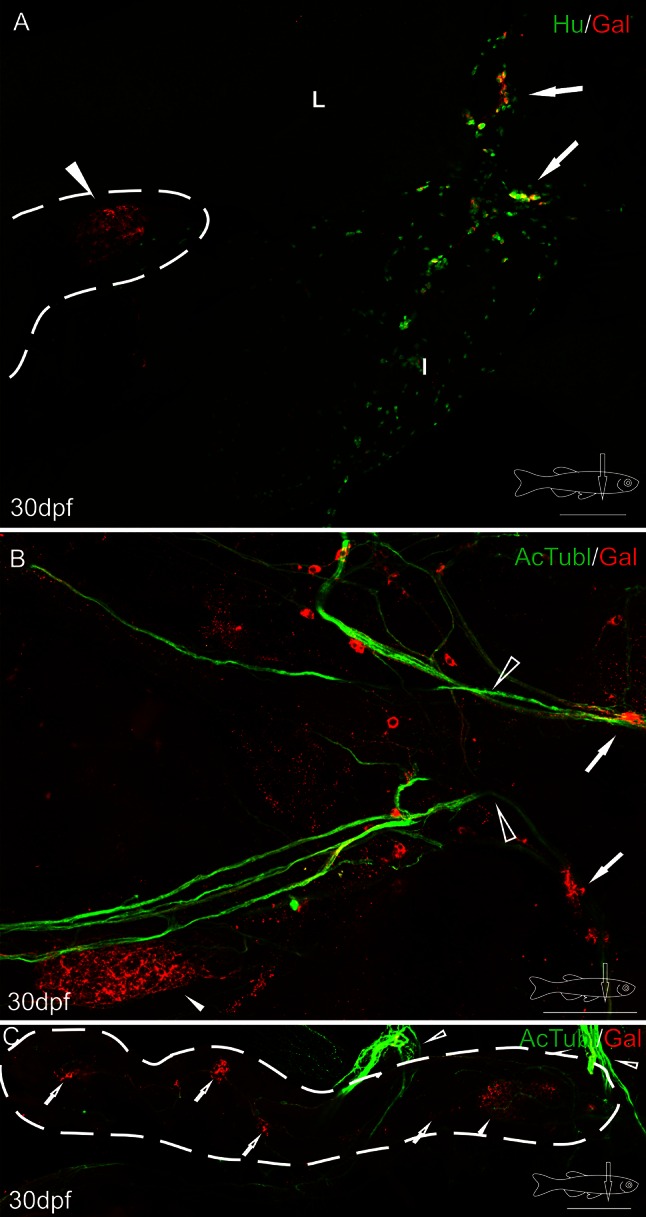
Whole-mount immunofluorescence staining of the 30 dpf zebrafish gut using antibody against galanin (**a**–**c**), neuronal marker Hu (**a**), and acetylated tubulin (**b**, **c**). *Arrows* point to galaninergic neurons in the autonomic ganglion, and the *arrowhead* shows a very dense network of galanin-IR fibers in the primary islet of the endocrine pancreas. The secondary islets (*hollow arrows*, **c**), the vagus nerve (*hollow arrowheads*, **b**, **c**) and its associated galanin-IR neurons are also visible. *Dashed line* indicates the pancreas (**a**, **c**). *I* intestine, *L* liver. *Scale bars* 100 µm

**Fig. 4:**
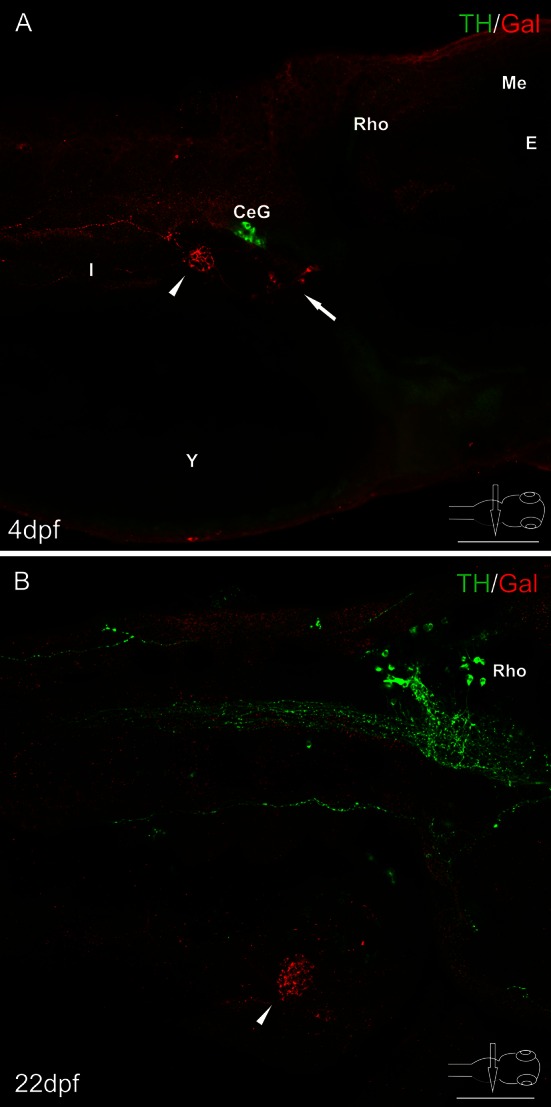
Whole-mount immunofluorescence staining of the 4 dpf zebrafish larvae (**a**), 22 dpf juvenile zebrafish (**b**) using antibody against galanin and adrenergic marker—tyrosine hydroxylase (TH). *Arrow* points to galaninergic neurons in autonomic ganglia. *Arrowheads* show the primary islet of the endocrine pancreas. Galanin-IR cells and fibers did not express immunoreactivity to TH. However, very intensely stained TH-IR neurons were found in the celiac ganglion (**a**) and in the brain (**b**). *CeG* celiac ganglion, *E* eye, *I* intestine, *Me* mesencephalon, *Rho* rhombencephalon. *Scale bars* 100 µm

**Fig. 5:**
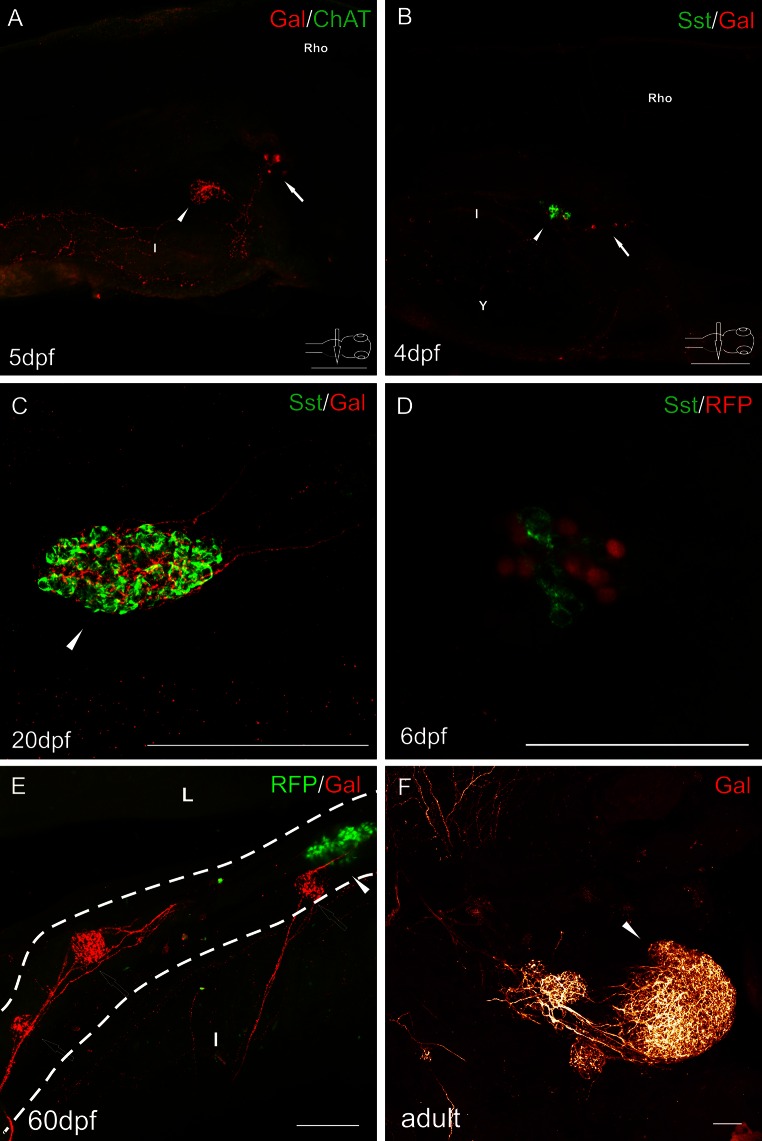
Whole-mount immunofluorescence staining of the zebrafish larvae (**a**, **b**, **d**), juvenile (**c**, **e**), and adult (**f**) guts using antibody against galanin (**a**–**c**, **e**–**f**), cholinergic marker—choline acetyltransferase (ChAT) (**a**), δ cell marker somatostatin (**b**–**d**). RFP marked mnx1+ population of β cells in the transgenic line of the zebrafish (**d**, **e**). *Arrows* point to galaninergic neurons in autonomic ganglia. *Arrowheads* show the primary islet of the endocrine pancreas, and hollow arrows point to the secondary islets. **a** There was no ChAT immunoreactivity in the cells and fibers associated with the pancreas and intestine. ChAT-IR neurons were observed only in the brain and spinal cord (not shown). Galanin-IR fibers were also visible, projecting from the autonomic ganglion to the intestine. **c**, **d** Pancreatic islets at a higher magnification. Somatostatin did not colocalize with β cell marker (**d**). Delta cells were very intensely supplied by galaninergic fibers (**c**, **d**). Note that the secondary islets did not contain RFP+ cells until 60 dpf (**e**). *Dashed line* indicates the pancreas. *I* intestine, *L* liver, *Rho* rhombencephalon. *Scale bars* 100 µm, except **d** = 50 µm

**Fig. 6:**
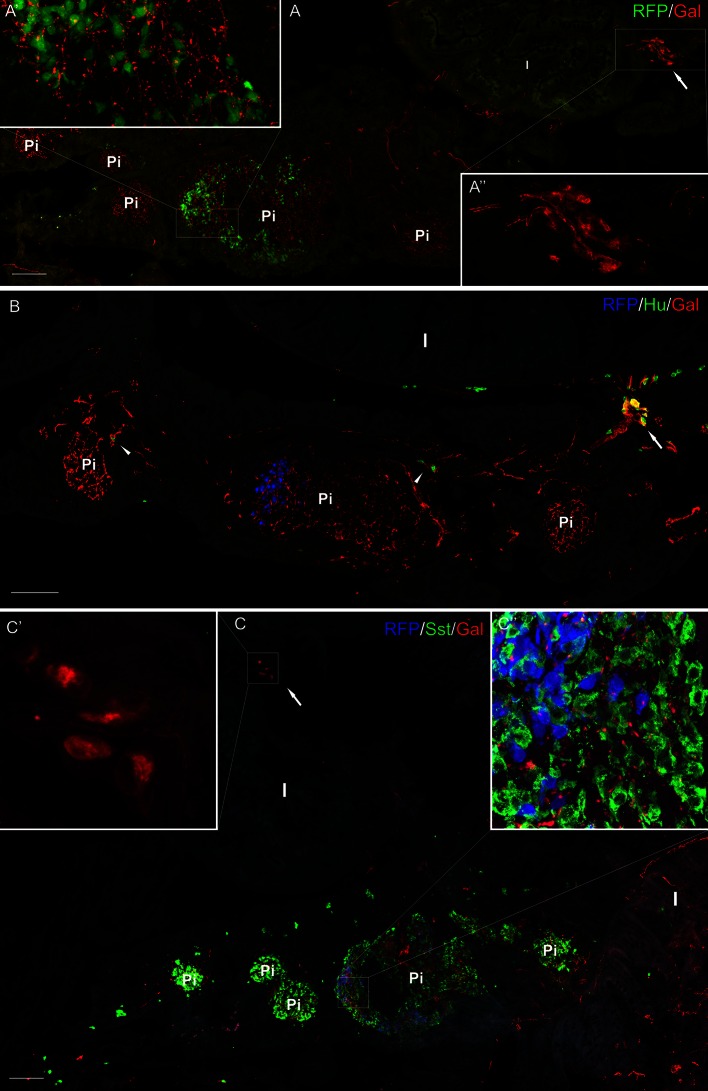
Section from the adult pancreas stained with antibody against galanin (**a**–**c**) and Hu (**b**), and somatostatin (**c**). RFP marked mnx1+ population of β cells in the transgenic line of the zebrafish (**a**–**c**). RFP+ cells were present mostly only in one (primary) islet, whereas somatostatin-positive cells were located in all the islets (**a**–**c**). Ganglion containing galanin-IR neurons is visible outside the pancreatic tissue, close to the intestinal wall (**a**, **a**″, **c**, **c**′). Neurons inside the pancreatic tissue are also visible; however, they were mostly galanin-negative (*arrowheads*, **b**). Galanin-IR fibers richly supplied pancreatic islets (**c**). *I* intestine, *Pi* pancreatic islet. *Scale bars* 100 µm

Immunofluorescent stainings revealed a very dense network of galanin-IR varicose fibers innervating the endocrine pancreas in the zebrafish in every developmental stage studied, from the second day post-fertilization (dpf; data not shown) to the adult fish (Figs. 2, 3, 4, 5, 6). The fibers were very densely packed with intensely stained galanin-immunoreactive (IR) vesicles (Figs. 2, 3, 4, 5, 6).

Confocal analysis of whole-mount galanin stainings in the larvae and juvenile fish revealed that the fibers originated from neurons located rostral to the pancreatic tissue (Figs. 2a, b, 3a, b, 4a, 5a, b). These galaninergic neurons were mostly clustered in two ganglia found bilaterally on both sides of the sagittal plane (Fig. 3a, b). The remaining galanin-IR neurons were distributed among galanin-negative nerve cell bodies forming large diffused ganglia (Figs. 2a, d, 3a). The ganglia were located on the dorsal side of the initial part of the anterior intestinal segment (Figs. 2b, 3a, 4a, 5a, b), close to the intestinal branch of the vagus nerve (Fig. 3b). The galanin-IR neurons usually formed strips of single cells distributed along the vagus nerve (Fig. 3b). Some of the galanin-IR neurons were scattered near the main ganglion (Figs. 2c, d, 3a, b). The small clusters of galanin-IR neurons and single perikarya were distributed mostly along ramifications of the vagus nerve, in particular at their branching points (Fig. 3b), which ran in the caudal direction toward the primary islet of the endocrine pancreas. The bundle of galanin-IR fibers supplied the primary islet and then ran into the caudal direction reaching next islets (Figs. 3c, 5e). From the 5 dpf, the galanin-IR nerve terminals richly innervated the intestine (Fig. 5a). In the adult fish, the galanin-IR ganglia were distributed in a way similar to that found in the juvenile animals, i.e., close to the anterior intestinal segment, outside the pancreatic tissue (Fig. 6). There were single neurons embedded in the pancreatic tissue; however, they rarely exhibited immunoreactivity to galanin (Fig. 6b). The neurons found within the pancreatic tissue were mostly small-sized cells (7–10 µm) and resembled morphologically enteric neurons, whereas the majority of galanin-IR neurons distributed outside the pancreas were larger in diameter (10–20 µm; Fig. 6).

In situ hybridization investigations with a probe for mRNA encoding galanin revealed groups of galaninergic cells in the same location which was occupied by neurons identified with the galanin antiserum (Fig. 1).

The galanin-IR neurons did not show immunoreactivity for applied antibody against tyrosine hydroxylase (TH), although TH-IR perikarya and nerve fibers were observed in the other parts of the central and peripheral nervous system (Fig. 4). Also, immunofluorescent investigations with antibodies against choline acetyltransferase (ChAT, Fig. 5a) or vesicular acetylcholine transporter (VAChT, data not shown) did not reveal immunoreactivity in the studied neurons. Interestingly, the antibodies against the cholinergic markers detected the appropriate immunoreactivities in perikarya and fibers in the brain and spinal cord, but not in nerve elements found outside the central nervous system.

Investigations with antibody against somatostatin (Sst) used on WT and Tg(mnx1:TagRFP) fish revealed immunoreactivity for this peptide in a large number of cells in all pancreatic islets (Figs. 5b–d, 6c). The marker of mnx1+ population of β cells (TagRFP) was found in a smaller number of cells in the primary islet (Figs. 2b, 5d, e, 6). In the secondary islets, single RFP+ cells were observed only occasionally. The secondary islets were also very densely supplied with galanin-IR fibers (Figs. 3c, 5e, 6). Moreover, the galanin-IR fibers more intensely innervated Sst+ cells than RFP+ cells in the primary islet (Fig. 6).

**Fig. 7:**
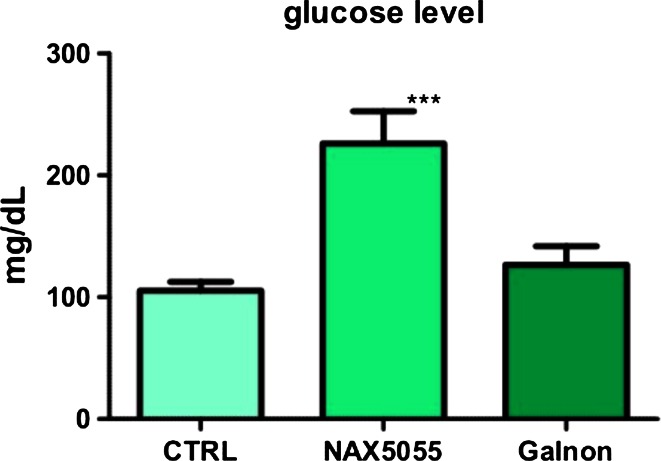
Effect of galanin receptor agonists (NAX 5055 and galnon) on blood glucose level. Results obtained in the NAX 5055-treated group were statistically significantly different from those obtained in the control group, *p* < 0.05. The level of blood glucose found after administration of galnon was not statistically significantly different from that observed in the control group, *p* > 0.05. One-way ANOVA with Tukey’s posttest. Data represent mean ± SE of the mean (*N* = 10)

Intraperitoneal injection of galanin analog NAX 5055 resulted in a statistically significant increase in the blood glucose level. The injection of another galanin receptor agonist, galnon, also caused a raise in the blood glucose level; however, it was not statistically significant (Fig. 7).

## Discussion

The present paper provides detailed information on galaninergic innervation of the zebrafish endocrine pancreas considering the source of the nerve fibers for the first time. Galaninergic innervation of the endocrine pancreas has been demonstrated in many species (see “Introduction” section). However, these studies have revealed prominent species-dependent differences dealing with both the density and possible sources of the galaninergic nerve supply.

In the dog, in which the effect of galanin on the blood glucose level was demonstrated for the first time (Tatemoto et al. 1983), galaninergic innervation of pancreatic islets is extensive (McDonald et al. 1992; Taborsky et al. 1999). Also in the mouse, the galanin-IR fibers are rather numerous (Lindskog et al. 1991). On the other hand, in the rat, pig, and humans, the endocrine pancreas is scarcely supplied by galaninergic nerves (McDonald et al. 1992; Lindskog et al. 1991). In our study, we have demonstrated that in the zebrafish, pancreatic islets receive very abundant galaninergic innervation. In other fish species, the galaninergic fibers are also quite numerous (Bosi et al. 2004; Putti et al. 2000). However, it seems that in the zebrafish, galaninergic innervation of pancreatic islets is much denser than in any animal species investigated so far.

The reason for such intense innervation in the zebrafish is not clear. One of the explanations could be that galanin plays a role as a trophic factor for the developing endocrine pancreas and this function of galanin has been well recognized (Hobson et al. 2010). However, the present results contradict this theory to some extent because the intense innervation was observed in each developmental stage studied and thus was also abundant in the adult animals.

The origin of galanin nerve fibers for the endocrine pancreas investigated previously in other species is not clear. There is a common view that in mammals, galanin is localized in the islet-projecting adrenergic/sympathetic nerve terminals (Ahren 2000). The source of these galaninergic fibers could be the celiac ganglion where galanin colocalizes with TH in some ganglionic neurons (Ahren et al. 1990). However, in at least certain species the galanin-IR nerve terminals have been found to be predominantly non-adrenergic, parasympathetic in nature (Verchere et al. 1996). Our study has revealed that sympathetic nerves are very rare or absent in the zebrafish islets and the galaninergic innervation of the endocrine pancreas is provided by neurons located in ganglia found close to the intestinal branch of the vagus nerve. Neurons in these ganglia do not contain TH which indicates that they do not belong to the sympathetic/adrenergic part of the autonomic nervous system.

The present study is the first to demonstrate non-adrenergic, non-sensory ganglia in the zebrafish and that these ganglia contain many galaninergic neurons projecting to the pancreas. The ganglia investigated most likely consist of postganglionic neurons derived from the vagal neural crest, and they are closely associated with the intestinal branches of the vagus nerve. It is tempting to assume that these ganglia, supplying the endocrine pancreas, are homologues of intrapancreatic/pancreatic ganglia described in mammals (Baltazar et al. 2001; Furuzawa et al. 1996; Myojin et al. 2000; Verchere et al. 1996) and other fish (Putti et al. 2000; Yui and Fujita 1986). They belong to the parasympathetic/cholinergic part of the autonomic nervous system; however, besides vagal innervation, their neurons receive sympathetic, enteric, and sensory supply (for review, see (Love et al. 2007). Most pancreatic neurons are ChAT-IR, which indicates they are cholinergic in nature (Liu et al. 1998). In our study, staining with antibodies against ChAT revealed no immunoreactivity in the ganglia. The antibody applied has been found to mark cholinergic structures very effectively in the zebrafish central nervous system (Castro et al. 2006; Yu et al. 2011). Also, in our investigations ChAT-IR neurons and fibers were visible in the larval brain and spinal cord. Other authors who performed immunostainings with the same ChAT antibody have reported lack of the IR structures in the peripheral nervous system in the juvenile stages of the zebrafish (Olsson et al. 2008; Uyttebroek et al. 2010). It is difficult to speculate about the reasons for the absence of immunoreactivity to ChAT in the ganglia studied. It is possible that the concentration of the antigens in the neurons is too low. Another explanation is that there is another isoform of the enzyme in the peripheral nervous system of the zebrafish larvae and the applied antibody recognizes only the isoform expressed in the central nervous system. This unrecognized isoform could be a counterpart of the peripheral type of ChAT (pChAT) described in mammals (Tooyama and Kimura 2000) or a product of duplicated ChAT gene (chatb) (Hong et al. 2013). The intrapancreatic ganglia, in addition to acetylcholine, express some neuropeptides including galanin (Baltazar et al. 2001; Furuzawa et al. 1996; Putti et al. 2000; Verchere et al. 1996), which confirms the present results.

Intraperitoneal injections of galanin analog NAX 5055 performed in our study resulted in an increase in blood glucose level in the adult zebrafish. Similar results have been obtained recently in the study on mice, where NAX 5055 produced a comparable effect (Flynn and White 2015). Our findings are also consistent with those of previous investigations which have revealed hyperglycemia in dogs after intravenous galanin infusion (Manabe et al. 1986; McDonald et al. 1985). Surprisingly, administration of galnon, a non-peptide galanin receptor agonist, had no significant influence on blood glucose level in the zebrafish. The lack of an apparent increase in blood glucose level after administration of galnon is consistent with the results of another study where galnon even stimulated insulin release in isolated rat pancreatic islets (Quynh et al. 2005). It has been shown that galnon has a moderate affinity to galanin receptors (Saar et al. 2002). It has multiple sites of interaction within the G protein-coupled receptor signaling cascade (Florén et al. 2005), and its effect is mediated by a site unrelated to galanin receptors which could explain differences in the influence on blood glucose level between galnon and NAX 5055.

The mechanism of galanin action on blood glucose level is not fully elucidated. However, there is a strong evidence that galanin inhibits insulin release from β cells of the endocrine pancreas (Flynn and White 2015; Gregersen et al. 1991; Lindskog and Ahren 1987; Lindskog et al. 1995). The inhibition of the insulin secretion by galanin has been postulated to be achieved by the reduction in intracellular cyclic AMP levels (Dunning et al. 1986; McDermott and Sharp 1995), direct modulation of the islet cell membrane potential (Amiranoff et al. 1988), inhibition of dihydropyridine-sensitive voltage-dependent L-type channels (Homaidan et al. 1991), and inhibition of islet exocytosis (Sharp et al. 1989). All three types of galanin receptors have been found in the pancreatic islets (Barreto et al. 2011). It can be assumed that an increase in blood glucose level observed in the present study after injection of NAX 5055 is mediated by galanin receptor 1 (GalR1), because NAX 5055 is a GalR1-preferring analog of galanin (Bulaj et al. 2008; White et al. 2009) and the expression of this receptor is the highest among other galanin receptors in the pancreatic islets (Barreto et al. 2011).

Galanin may act directly on the β cells and inhibit insulin release. The present study has revealed a very dense network of galanin-IR fibers supplying pancreatic islets in the zebrafish. However, the β cells visualized in Tg(mnx1:TagRFP) zebrafish line with RFP were localized only in the primary islet. On the other hand, the intense galaninergic innervation was observed in both the primary and secondary islets. As mentioned, Wendik et al. (2004) have reported that in the zebrafish pancreas, *mnx1* is expressed exclusively in β cells by 20 hpf. However, Maddison and Chen (2012) have found that in the animals of Tg(mnx1:TagRFP) line, not all the β cells express RFP. It is thus possible that galaninergic fibers observed in our study in the secondary islets also innervate β cells which were RFP-negative. It can also be assumed that galanin influences blood glucose level indirectly and the main target of the galaninergic fibers could also be the population of the δ cells because a close association between galanin-IR nerve terminals and the somatostatin-expressing cells was apparent. Thus, the effect on blood glucose level could be achieved through the involvement of somatostatin. It has been found that galanin exerts inhibitory effect on the somatostatin release from the pancreas (Dunning et al. 1986; Boyle et al. 1994). Several studies indicate that somatostatin is a physiologically important paracrine factor (de Heer et al. 2008; Hauge-Evans et al. 2009) involved in the regulation of insulin and glucagon release from the pancreatic islets (Hauge-Evans et al. 2009). This peptide may exert a tonic inhibitory influence on insulin and glucagon secretion, which may facilitate the islet response to cholinergic activation. It is also possible that galanin influences blood glucose level through other pathways because some studies indicate that the peptide stimulates glucagon secretion (Boyle et al. 1994; Dunning et al. 1986; Lindskog and Ahren 1987). However, the exact mechanism of galanin action on the pancreatic hormone production and/or secretion should be elucidated by further studies.

In conclusion, the present study has disclosed the existence of non-adrenergic, non-sensory ganglia in the zebrafish for the first time. They provide an abundant innervation to the endocrine pancreas and to other viscera. Neurons in these ganglia express neuropeptide galanin which regulates blood glucose level, a phenomenon also found in other species investigated. The present study has also demonstrated that administration of galanin analog NAX 5055 causes an increase in the blood glucose level in the zebrafish. The present findings suggest that, like in mammals, in the zebrafish galanin is involved in the regulation of blood glucose level. However, further studies are needed to elucidate the exact mechanism of the galanin action.

## References

[CR1] Adeghate E, Ponery AS (2001). Large reduction in the number of galanin-immunoreactive cells in pancreatic islets of diabetic rats. J Neuroendocrinol.

[CR2] Adeghate E, Ember Z, Donáth T, Pallot DJ, Singh J (1996). Immunohistochemical identification and effects of atrial natriuretic peptide, pancreastatin, leucine-enkephalin, and galanin in the porcine pancreas. Peptides.

[CR3] Ahren B (2000). Autonomic regulation of islet hormone secretion—implications for health and disease. Diabetologia.

[CR4] Ahrén B (1990). Effects of galanin and calcitonin gene-related peptide on insulin and glucagon secretion in man. Acta Endocrinol.

[CR5] Ahren B, Taborsky GJ, Porte D (1986). Neuropeptidergic versus cholinergic and adrenergic regulation of islet hormone secretion. Diabetologia.

[CR6] Ahren B, Bottcher G, Kowalyk S, Dunning BE, Sundler F, Taborsky GJ (1990). Galanin is co-localized with noradrenaline and neuropeptide Y in dog pancreas and celiac ganglion. Cell Tissue Res.

[CR7] Ahren B, Ar’Rajab A, Bottcher G, Sundler F, Dunning BE (1991). Presence of galanin in human pancreatic nerves and inhibition of insulin secretion from isolated human islets. Cell Tissue Res.

[CR8] Amiranoff B, Lorinet AM, Lagny-Pourmir I, Laburthe M (1988). Mechanism of galanin-inhibited insulin release. Occurrence of a pertussis-toxin-sensitive inhibition of adenylate cyclase. Eur J Biochem.

[CR9] Baltazar ET, Kitamura N, Hondo E, Narreto EC, Yamada J (2000). Galanin-like immunoreactive endocrine cells in bovine pancreas. J Anat.

[CR10] Baltazar ET, Kitamura N, Sasaki M, Cottrell DF, Boloron HM, Yamada J (2001). Galanin-like immunoreactive neural elements in domestic ruminant pancreas. J Vet Med Sci.

[CR11] Barreto SG, Bazargan M, Zotti M, Hussey DJ, Sukocheva OA, Peiris H, Leong M, Keating DJ, Schloithe AC, Carati CJ, Smith C, Toouli J, Saccone GTP (2011). Galanin receptor 3—a potential target for acute pancreatitis therapy. Neurogastroenterol Motil.

[CR12] Bosi G, Di Giancamillo A, Arrighi S, Domeneghini C (2004). An immunohistochemical study on the neuroendocrine system in the alimentary canal of the brown trout, *Salmo trutta*, L., 1758. Gen Comp Endocrinol.

[CR13] Bosi G, Bermudez R, Domeneghini C (2007). The galaninergic enteric nervous system of pleuronectiformes (Pisces, Osteichthyes): an immunohistochemical and confocal laser scanning immunofluorescence study. Gen Comp Endocrinol.

[CR14] Boyle MR, Verchere CB, McKnight G, Mathews S, Walker K, Taborsky GJ (1994). Canine galanin: sequence, expression and pancreatic effects. Regul Pept.

[CR15] Brown LA, Stoskopf MK (1993). Anesthesia and restraint. Fish medicine.

[CR16] Brunicardi FC, Shavelle DM, Andersen DK (1995). Neural regulation of the endocrine pancreas. Int J Pancreatol.

[CR17] Bulaj G, Green BR, Lee HK, Robertson CR, White K, Zhang L, Sochanska M, Flynn SP, Scholl EA, Pruess TH, Smith MD, White HS (2008). Design, synthesis, and characterization of high-affinity, systemically-active galanin analogues with potent anticonvulsant activities. J Med Chem.

[CR18] Castro A, Becerra M, Manso MJ, Anadon R (2006). Calretinin immunoreactivity in the brain of the zebrafish, *Danio rerio*: distribution and comparison with some neuropeptides and neurotransmitter-synthesizing enzymes. I. Olfactory organ and forebrain. J Comp Neurol.

[CR19] Ch’ng JL, Christofides ND, Anand P, Gibson SJ, Allen YS, Su HC, Tatemoto K, Morrison JF, Polak JM, Bloom SR (1985). Distribution of galanin immunoreactivity in the central nervous system and the responses of galanin-containing neuronal pathways to injury. Neuroscience.

[CR20] Chen YC, Priyadarshini M, Panula P (2009). Complementary developmental expression of the two tyrosine hydroxylase transcripts in zebrafish. Histochem Cell Biol.

[CR21] de Heer J, Rasmussen C, Coy DH, Holst JJ (2008). Glucagon-like peptide-1, but not glucose-dependent insulinotropic peptide, inhibits glucagon secretion via somatostatin (receptor subtype 2) in the perfused rat pancreas. Diabetologia.

[CR22] Della Rossa A, Putti R (1995). The endocrine pancreas of lacertids: an immunocytochemical study of the genera Pedioplanis and Meroles. Eur J Histochem.

[CR23] Dunning BE, Ahren B, Veith RC, Bottcher G, Sundler F, Taborsky GJ (1986). Galanin: a novel pancreatic neuropeptide. Am J Physiol.

[CR24] Eames SC, Philipson LH, Prince VE, Kinkel MD (2010). Blood sugar measurement in zebrafish reveals dynamics of glucose homeostasis. Zebrafish.

[CR25] Florén A, Sollenberg U, Lundström L, Zorko M, Stojan J, Budihna M, Wheatley M, Martin NP, Kilk K, Mazarati A, Bartfai T, Lindgren M, Langel Ü (2005). Multiple interaction sites of galnon trigger its biological effects. Neuropeptides.

[CR26] Flynn SP, White HS (2015). Regulation of glucose and insulin release following acute and repeated treatment with the synthetic galanin analog NAX-5055. Neuropeptides.

[CR27] Furuzawa Y, Ohmori Y, Watanabe T (1996). Immunohistochemical studies of neural elements in pancreatic islets of the cat. J Vet Med Sci.

[CR28] Gilbey SG, Stephenson J, O’Halloran DJ, Burrin JM, Bloom SR (1989). High-dose porcine galanin infusion and effect on intravenous glucose tolerance in humans. Diabetes.

[CR29] Gregersen S, Hermansen K, Langel U, Fisone G, Bartfai T, Ahren B (1991). Galanin-induced inhibition of insulin secretion from rat islets: effects of rat and pig galanin and galanin fragments and analogues. Eur J Pharmacol.

[CR30] Hauge-Evans AC, King AJ, Carmignac D, Richardson CC, Robinson IC, Low MJ, Christie MR, Persaud SJ, Jones PM (2009). Somatostatin secreted by islet delta-cells fulfills multiple roles as a paracrine regulator of islet function. Diabetes.

[CR31] Hiramatsu K, Ohshima K (1995). Immunohistochemical study on the distribution of galanin-containing nerves in the chicken pancreas. Histol Histopathol.

[CR32] Hobson SA, Bacon A, Elliot-Hunt CR, Holmes FE, Kerr NC, Pope R, Vanderplank P, Wynick D (2010). Galanin acts as a trophic factor to the central and peripheral nervous systems. Exp Suppl.

[CR33] Hokfelt T, Xu ZQD, Shi TJ, Holmberg K, Zhang X (1998). Galanin in ascending systems—focus on coexistence with 5-hydroxytryptamine and noradrenaline. Ann NY Acad Sci.

[CR34] Homaidan FR, Sharp GW, Nowak LM (1991). Galanin inhibits a dihydropyridine-sensitive Ca^2+^ current in the RINm5f cell line. Proc Natl Acad Sci USA.

[CR35] Hong E, Santhakumar K, Akitake CA, Ahn SJ, Thisse C, Thisse B, Wyart C, Mangin JM, Halpern ME (2013). Cholinergic left–right asymmetry in the habenulo-interpeduncular pathway. Proc Natl Acad Sci USA.

[CR36] Iwama GK, Ackerman PA, Hochachka PW, Mommsen TP (1994). Anaesthetics. Analytical techniques.

[CR37] Jao LE, Appel B, Wente SR (2012). A zebrafish model of lethal congenital contracture syndrome 1 reveals Gle1 function in spinal neural precursor survival and motor axon arborization. Development.

[CR38] Jopling C, Sleep E, Raya M, Marti M, Raya A, Belmonte JCI (2010). Zebrafish heart regeneration occurs by cardiomyocyte dedifferentiation and proliferation. Nature.

[CR39] Kawahara G, Karpf JA, Myers JA, Alexander MS, Guyon JR, Kunkel LM (2011). Drug screening in a zebrafish model of Duchenne muscular dystrophy. Proc Natl Acad Sci.

[CR40] Kawakami T, Kusakabe T, Takenaka T (1995). Distribution of immunoreactive neuropeptides in the pancreas of the bullfrog, *Rana catesbeiana*, demonstrated by immunofluorescence. Cell Tissue Res.

[CR41] Kinkel MD, Prince VE (2009). On the diabetic menu: zebrafish as a model for pancreas development and function. Bioessays.

[CR42] Lindskog S, Ahren B (1987). Galanin: effects on basal and stimulated insulin and glucagon secretion in the mouse. Acta Physiol Scand.

[CR43] Lindskog S, Ahren B, Dunning BE, Sundler F (1991). Galanin-immunoreactive nerves in the mouse and rat pancreas. Cell Tissue Res.

[CR44] Lindskog S, Gregersen S, Hermansen K, Ahren B (1995). Effects of galanin on proinsulin mRNA and insulin biosynthesis in normal islets. Regul Pept.

[CR45] Liu HP, Tay SS, Leong S, Schemann M (1998). Colocalization of ChAT, DbetaH and NADPH-d in the pancreatic neurons of the newborn guinea pig. Cell Tissue Res.

[CR46] Love JA, Yi E, Smith TG (2007). Autonomic pathways regulating pancreatic exocrine secretion. Auton Neurosci.

[CR47] Maddison LA, Chen W (2012). Nutrient excess stimulates beta-cell neogenesis in zebrafish. Diabetes.

[CR48] Manabe T, Yoshimura T, Kii E, Tanaka Y, Ohshio G, Tobe T, Akaji K, Yajima H (1986). Galanin-induced hyperglycemia: effect on insulin and glucagon. Endocr Res.

[CR49] Mazarati AM, Hohmann JG, Bacon A, Liu H, Sankar R, Steiner RA, Wynick D, Wasterlain CG (2000). Modulation of hippocampal excitability and seizures by galanin. J Neurosci.

[CR50] McDermott AM, Sharp GWG (1995). Gi2 and Gi3 proteins mediate the inhibition of adenylyl cyclase by galanin in the RINm5F cell. Diabetes.

[CR51] McDonald TJ, Dupre J, Tatemoto K, Greenberg GR, Radziuk J, Mutt V (1985). Galanin inhibits insulin secretion and induces hyperglycemia in dogs. Diabetes.

[CR52] McDonald TJ, Brooks BD, Rokaeus A, Tinner B, Staines WA (1992). Pancreatic galanin: molecular forms and anatomical locations. Pancreas.

[CR53] Melander T, Hokfelt T, Rokaeus A (1986). Distribution of galaninlike immunoreactivity in the rat central nervous system. J Comp Neurol.

[CR54] Messell T, Harling H, Böttcher G, Johnsen AH, Holst JJ (1990). Galanin in the porcine pancreas. Regul Pept.

[CR55] Miller RE (1981). Pancreatic neuroendocrinology: peripheral neural mechanisms in the regulation of the islets of langerhans. Endocr Rev.

[CR56] Misane I, Razani H, Wang FH, Jansson A, Fuxe K, Ogren SO (1998). Intraventricular galanin modulates a 5-HT1A receptor-mediated behavioural response in the rat. Eur J Neurosci.

[CR57] Myojin T, Kitamura N, Hondo E, Baltazar ET, Pearson GT, Yamada J (2000). Immunohistochemical localization of neuropeptides in bovine pancreas. Anat Histol Embryol.

[CR58] Nilsson T, Arkhammar P, Rorsman P, Berggren P- (1989). Suppression of insulin release by galanin and somatostatin is mediated by a G-protein. An effect involving repolarization and reduction in cytoplasmic free Ca^2+^ concentration. J Biol Chem.

[CR59] Olsen AS, Sarras MP, Intine RV (2010). Limb regeneration is impaired in an adult zebrafish model of diabetes mellitus. Wound Repair Regen.

[CR60] Olsson C, Holmberg A, Holmgren S (2008). Development of enteric and vagal innervation of the zebrafish (*Danio rerio*) gut. J Comp Neurol.

[CR61] Panula P, Sallinen V, Sundvik M, Kolehmainen J, Torkko V, Tiittula A, Moshnyakov M, Podlasz P (2006). Modulatory neurotransmitter systems and behavior: towards zebrafish models of neurodegenerative diseases. Zebrafish.

[CR62] Pieribone VA, Xu ZQ, Zhang X, Grillner S, Bartfai T, Hokfelt T (1995). Galanin induces a hyperpolarization of norepinephrine-containing locus-coeruleus neurons in the brain-stem slice. Neuroscience.

[CR63] Podlasz P, Sallinen V, Chen Y-, Kudo H, Fedorowska N, Panula P (2012). Galanin gene expression and effects of its knock-down on the development of the nervous system in larval zebrafish. J Comp Neurol.

[CR64] Proshchina AE, Krivova YS, Barabanov VM, Saveliev SV (2014). Ontogeny of neuro-insular complexes and islets innervation in the human pancreas. Front Endocrinol.

[CR65] Putti R, Maglio M, Odierna G (2000). An immunocytochemical study of intrapancreatic ganglia, nerve fibres and neuroglandular junctions in Brockmann bodies of the tompot blenny (*Blennius gattoruggine*), a marine teleost. Histochem J.

[CR66] Quynh NT, Islam MS, Floren A, Bartfai T, Langel U, Ostenson CG (2005). Effects of galnon, a non-peptide galanin-receptor agonist, on insulin release from rat pancreatic islets. Biochem Biophys Res Commun.

[CR67] Saar K, Mazarati AM, Mahlapuu R, Hallnemo G, Soomets U, Kilk K, Hellberg S, Pooga M, Tolf B, Shi TS, Hökfelt T, Wasterlain C, Bartfai T, Langel Ü (2002). Anticonvulsant activity of a nonpeptide galanin receptor agonist. Proc Natl Acad Sci.

[CR68] Santoriello C, Gennaro E, Anelli V, Distel M, Kelly A, Köster RW, Hurlstone A, Mione M (2010). Kita driven expression of oncogenic HRAS leads to early onset and highly penetrant melanoma in zebrafish. PLoS One.

[CR69] Sharp GW, Le Marchand-Brustel Y, Yada T, Russo LL, Bliss CR, Cormont M, Monge L, Van Obberghen E (1989). Galanin can inhibit insulin release by a mechanism other than membrane hyperpolarization or inhibition of adenylate cyclase. J Biol Chem.

[CR70] Shimosegawa T, Moriizumi S, Koizumi M, Kashimura J, Yanaihara N, Toyota T (1992). Immunohistochemical demonstration of galaninlike immunoreactive nerves in the human pancreas. Gastroenterology.

[CR71] Skofitsch G, Jacobowitz DM (1985). Galanin-like immunoreactivity in capsaicin sensitive sensory neurons and ganglia. Brain Res Bull.

[CR72] Swanhart LM, Cosentino CC, Diep CQ, Davidson AJ, de Caestecker M, Hukriede NA (2011). Zebrafish kidney development: basic science to translational research. Birth Defects Res Part C Embryo Today Rev.

[CR73] Taborsky GJ, Dunning BE, Havel PJ, Ahren B, Kowalyk S, Boyle MR, Verchere CB, Baskin DG, Mundinger TO (1999). The canine sympathetic neuropeptide galanin: a neurotransmitter in pancreas, a neuromodulator in liver. Horm Metab Res.

[CR74] Tatemoto K, Rökaeus Å, Jörnvall H, McDonald TJ, Mutt V (1983). Galanin—a novel biologically active peptide from porcine intestine. FEBS Lett.

[CR75] Tooyama I, Kimura H (2000). A protein encoded by an alternative splice variant of choline acetyltransferase mRNA is localized preferentially in peripheral nerve cells and fibers. J Chem Neuroanat.

[CR76] Uyttebroek L, Shepherd IT, Harrisson F, Hubens G, Blust R, Timmermans JP, Van Nassauw L (2010). Neurochemical coding of enteric neurons in adult and embryonic zebrafish (*Danio rerio*). J Comp Neurol.

[CR77] Verchere CB, Kowalyk S, Koerker DJ, Baskin DG, Taborsky GJ (1996). Evidence that galanin is a parasympathetic, rather than a sympathetic, neurotransmitter in the baboon pancreas. Regul Pept.

[CR78] Wendik B, Maier E, Meyer D (2004). Zebrafish mnx genes in endocrine and exocrine pancreas formation. Dev Biol.

[CR79] Westerfield M (2000). The zebrafish book: a guide for the laboratory use of zebrafish (*Danio rerio*).

[CR80] White HS, Scholl EA, Klein BD, Flynn SP, Pruess TH, Green BR, Zhang L, Bulaj G (2009). Developing novel antiepileptic drugs: characterization of NAX 5055, a systemically-active galanin analog, in epilepsy models. Neurotherapeutics.

[CR81] Xi Y, Noble S, Ekker M (2011). Modeling neurodegeneration in zebrafish. Curr Neurol Neurosci Rep.

[CR82] Yu YM, Cristofanilli M, Valiveti A, Ma L, Yoo M, Morellini F, Schachner M (2011). The extracellular matrix glycoprotein tenascin-C promotes locomotor recovery after spinal cord injury in adult zebrafish. Neuroscience.

[CR83] Yui R, Fujita T (1986). Immunocytochemical studies on the pancreatic islets of the ratfish *Chimaera monstrosa*. Arch Histol Jpn.

[CR84] Zini S, Roisin MP, Langel U, Bartfai T, Benari Y (1993). Galanin reduces release of endogenous excitatory amino-acids in the rat hippocampus. Eur J Pharmacol Mol Pharmacol Sect.

